# Recognizing protected and anthropogenic patterns in landscapes using interpretable machine learning and satellite imagery

**DOI:** 10.3389/frai.2023.1278118

**Published:** 2023-12-01

**Authors:** Timo T. Stomberg, Johannes Leonhardt, Immanuel Weber, Ribana Roscher

**Affiliations:** ^1^Remote Sensing Group, Institute of Geodesy and Geoinformation, Faculty of Agriculture, University of Bonn, Bonn, Germany; ^2^AB–EX Community, PLEdoc GmbH, Essen, Germany; ^3^Data Science for Crop Systems, Institute of Bio- and Geosciences, Plant Sciences, Forschungszentrum Jülich GmbH, Jülich, Germany

**Keywords:** explainable machine learning, attributions, saliency maps, weakly-supervised learning, remote sensing, Sentinel-2, territorial protection, AnthroProtect

## Abstract

The accurate and comprehensive mapping of land cover has become a central task in modern environmental research, with increasing emphasis on machine learning approaches. However, a clear technical definition of the land cover class is a prerequisite for learning and applying a machine learning model. One of the challenging classes is naturalness and human influence, yet mapping it is important due to its critical role in biodiversity conservation, habitat assessment, and climate change monitoring. We present an interpretable machine learning approach to map patterns related to territorial protected and anthropogenic areas as proxies of naturalness and human influence using satellite imagery. To achieve this, we train a weakly-supervised convolutional neural network and subsequently apply attribution methods such as Grad-CAM and occlusion sensitivity mapping. We propose a novel network architecture that consists of an image-to-image network and a shallow, task-specific head. Both sub-networks are connected by an intermediate layer that captures high-level features in full resolution, allowing for detailed analysis with a wide range of attribution methods. We further analyze how intermediate layer activations relate to their attributions across the training dataset to establish a consistent relationship. This makes attributions consistent across different scenes and allows for a large-scale analysis of remote sensing data. The results highlight that our approach is a promising way to observe and assess naturalness and territorial protection.

## 1 Introduction

Accurate and comprehensive land cover (LC) mapping has become a central task in modern environmental research, driven by the increasing adoption of machine learning (ML) methods. However, this process faces challenges, particularly in defining and classifying LC types that lack a clear technical definition, such as natural and human-influenced areas. Nevertheless, accurate mapping of these regions is crucial, as areas with limited human influence offer significant ecological and social benefits. Therefore, it is important to identify and preserve such positive characteristics and ecological functions for conservation and sustainable development. Approaches such as the human influence index by Sanderson et al. (2002) provide important insights into the global distribution of naturalness. However, they are restricted to low spatial and temporal resolution due to their underlying data. In contrast, satellite imagery is a valuable tool for continuously monitoring undisturbed regions, moreover reducing the need for physical visits. To address the ambiguity in mapping ecological naturalness and move toward a more technical characterization usable for automated mapping, interpretable ML techniques can be used, as highlighted by Roscher et al. (2020).

Interpretable ML refers to the development of models and techniques that represent the ML decision process in understandable terms to humans. When combined with domain knowledge, these interpretations can lead to explanations that are sought for various reasons. Beyond gaining trust as a common reason, interpretable ML supports finding patterns in the given data and thus can provide novel scientific knowledge. Attribution methods, for example, are valuable tools to detect patterns in images by assigning an attribution value to each pixel of the input image. This can be achieved, for example, by training a convolutional neural network (CNN) using targets at an image level, and, subsequently, extracting information at a pixel level. Such a strategy can be seen as a weakly-supervised segmentation task, where the attributions are used to pixel-wise decide on the semantic class (Kwak et al., 2017; Selvaraju et al., 2020; Zhang and Ma, 2021).

**Figure 1:**
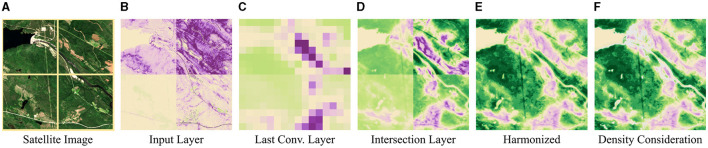
Grad-CAM applied to different NN layers and harmonized Grad-CAM attributions. **(A)** Satellite image. The image is tiled into four tiles with 256 × 256 pixels each. Each tile is treated separately for the following experimental examples. **(B)** Grad-CAM applied to the input layer. The attributions consider low-level features, only. **(C)** Grad-CAM applied to the last convolutional layer. High-level features are considered but the resolution is low. **(D)** Grad-CAM applied to our intermediate layer. High-level features are considered in full resolution. However, attributions exhibit inconsistency across the tiles resulting in various intensities. **(E)** Harmonized Grad-CAM attributions. Attributions are consistent. **(F)** Harmonized attributions with density consideration. Activations that are rarely represented in the training data are not considered when determining the attributions and are masked (gray color). [Copernicus Sentinel data 2020].

In this article, we introduce a method to map patterns that account for protected and anthropogenic areas, as proxies for naturalness and human influence, respectively. To this end, we use satellite images from such regions to train a CNN with targets at an image level. Subsequently, we employ diverse attribution methods and map the patterns related to the two categories to test scenes. In doing so, we propose a novel CNN architecture that consists of an image-to-image network and a shallow, task-specific head, e.g., a regressor or classifier. The intermediate layer thus represents the patterns within the input as high-level features with regard to the task at hand in full resolution. The attributions of this representation are determined at a pixel-wise level by applying a desired attribution method to the NN head. We provide two methodological novelties concerning the NN architecture and the evaluation of attributions:

- We provide high-level attributions with the resolution of the input image (Figures 1A, D).
- We harmonize the attributions consistently across similar activations throughout the training dataset. This way, we establish a direct linkage between activations and attributions that can be used for mapping test scenes (Figure 1E).

## 2 Related work

### 2.1 Mapping naturalness

The human influence index by Sanderson et al. (2002) provides important insights into the global distribution of natural areas. It is based on population density, land transformation, accessibility indicators such as roads, and electrical power infrastructure. Defined scoring methods result in an index, globally mapped with a resolution of 1 km^2^. The wilderness quality index by Fisher et al. (2010) is mapped within Europe using similar databases, but terrain ruggedness is used instead of electrical power infrastructure. Both methods are restricted to the low spatial and temporal resolution of the data they are based on. Replacing some of the reliant indicators with more recent geospatial data, the naturalness index by Ekim et al. (2021) achieves a resolution of 10 m for regional maps. However, these mapping approaches offer limited insight since they are based on human-made assumptions. Using remote sensing data to recognize patterns found in protected areas using machine learning can solve this issue. Ma et al. (2019) review the variety of remote sensing applications that can be addressed with deep learning including a critical conclusion and open challenges. Several remote sensing applications have been used for monitoring protected areas (Wang et al., 2020).

### 2.2 Explainable ML and attribution methods

ML models are able to find patterns and relations in large datasets that are not necessarily recognizable by humans. Roscher et al. (2020) highlight the usefulness of such models being interpretable and explainable. There are various approaches in the field of explainable ML, where Samek et al. (2021) review the important ones specifically for deep learning applications. For example, attribution methods such as Grad-CAM proposed by Selvaraju et al. (2020) and occlusion sensitivity mapping proposed by Zeiler and Fergus (2014) are valuable tools to detect image patterns by assigning an attribution value to each pixel of the input image.

There are various methods for achieving attribution maps. Gradient-based approaches such as saliency maps by Simonyan et al. (2014) take the gradients of the output score regarding the input image as a measure for attribution. Since saliency maps tend to be noisy, there are numerous extensions to this approach such as SmoothGrad by Smilkov et al. (2017), Guided Backpropagation by Springenberg et al. (2015), and Integrated Gradients by Sundararajan et al. (2017). For Grad-CAM (Gradient-weighted Class Activation Mapping), gradients are usually computed regarding the last convolutional layer, averaged, and multiplied by the activations of this layer (Selvaraju et al., 2020). A different approach to receiving attribution maps is to occlude parts in the input image and to identify subsequent changes in the model's outcome. Zeiler and Fergus (2014) replace similarly shaped patches with a specific value in the input image to compute occlusion sensitivity maps. Petsiuk et al. (2018) extend this approach by occluding random areas in the image instead of uniformly shaped patches (Randomized Input Sampling for Explanation, RISE). There are multiple other well-known attribution methods, such as LIME (Local Interpretable Model-agnostic Explanations) by Ribeiro et al. (2016) and SHAP (SHapley Additive exPlanations) by Lundberg and Lee (2017). Several ones are evaluated on models for LC classification using Sentinel data by Kakogeorgiou and Karantzalos (2021). They conclude that the approaches Grad-CAM, occlusions, and LIME lead to the most reliable results.

Most CNNs handling image-wise targets are built such that, as the network gets deeper, the layers have a higher number of channels but a lower resolution. This way, the receptive field of the filters becomes larger and at the same time, fewer computations have to be performed. Bengio et al. (2013), Zeiler and Fergus (2014), and Selvaraju et al. (2020) show, that the learned representation of a CNN captures higher-level features in deeper layers. As a consequence, attribution methods applied to deep convolutional layers consider high-level semantics but the resulting attribution maps have a low resolution (cf. Figure 1C). On the other hand, if applied to the input image low-level semantics are considered in full resolution (Figure 1B). Adebayo et al. (2020) show in their experiments that, in this case, many saliency methods have an analogy to edge detectors only. Therefore, Selvaraju et al. (2020) recommend applying Grad-CAM to the last convolutional layer and increasing the resolution by bilinear upsampling.

Attribution methods provide insights into the model's decision for individual input images. Nevertheless, the presence of multiple patterns within a single image introduces a complexity where these patterns can interact and influence each other. Consequently, similar patterns might exhibit diverse attributions across different images. However, especially in the field of remote sensing, it is important to universally evaluate attribution maps of large scenes and to be able to compare them with each other. Our idea for harmonizing attributions builds upon the approach of Stomberg et al. (2021), who utilize a similar network architecture relating activations to specific concepts.

## 3 Methodology

### 3.1 Network architecture and training

**Figure 2:**
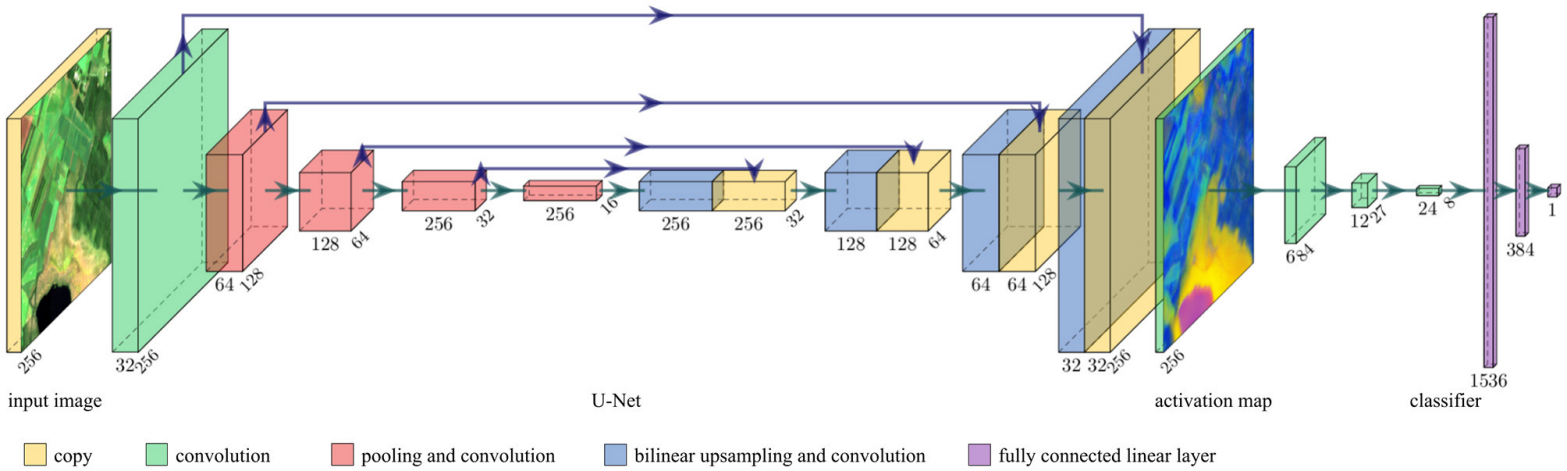
Architecture of our NN. It consists of a modified U-Net, followed by a task-specific head (regressor or classifier). It takes multispectral images and predicts one score ŷ∈(0, 1). The activation map at the intermediate layer has the same height *h* and width *w* as the input image. [Created with PlotNeuralNet by Iqbal (2018); Copernicus Sentinel data 2020].

To obtain high-level features in full resolution, we combine an image-to-image NN and a task-specific head. For the experiments shown in this article we use a modified form of the U-Net by Ronneberger et al. (2015), as it is a well-known architecture for image-to-image tasks; and a task-specific head (regressor or classifier) with significantly fewer parameters consisting of convolutional and linear layers (see Figure 2).

Our modified U-Net has the following characteristics: (1) It consists of four encoding and four decoding steps. Instead of two convolutional layers per encoding or decoding step, our U-Net has only one such layer. Furthermore, we reduce the number of output channels for each convolution by a factor of two. It turns out that the so reduced number of parameters is sufficient for the complexity of our task. (2) The convolutional layers are activated with leaky ReLU. ReLU could be used just as well. (3) We add batch normalization after each convolutional layer. (4) Including padding to each convolution, we preserve the image size at each skip connection. (5) We replace the deconvolutional upsampling with bilinear upsampling as proposed by Odena et al. (2016) to prevent checkerboard artifacts. (6) Instead of a single-channel input image, our U-Net takes *C*_in_-channel input images. Furthermore, we consider the number of activation map channels *C*_act_ as a hyperparameter. The activation map is the output of the U-Net. (7) The activation map is not batch-normalized and activated with the hyperbolic tangent function (tanh) so that the activation map has values in the range of −1 and 1. With this architecture, the predicted activation map of an image with shape (*h*, *w*, *C*_in_) has shape (*h*, *w*, *C*_act_)—so height *h* and width *w* remain unchanged.

The task-specific head consists of three convolutional layers and two fully connected linear layers. Each convolutional layer doubles the number of channels, has a kernel size of 5, and a stride of 3. The output of the last convolutional layer is flattened. Two fully connected linear layers follow with 384 and 1 neuron(s), respectively. All hidden layers are activated with leaky ReLU.

The U-Net and the task-specific head are trained jointly end-to-end on a regression or classification task. The loss function and final activation function are chosen according to the task (see Section 5.1).

### 3.2 Attribution methods and harmonization

After the NN has been trained, a desired attribution method is applied to the intermediate layer using the task-specific head. This way, attributions of the representing high-level features are determined on a pixel-wise level.

**Figure 3:**
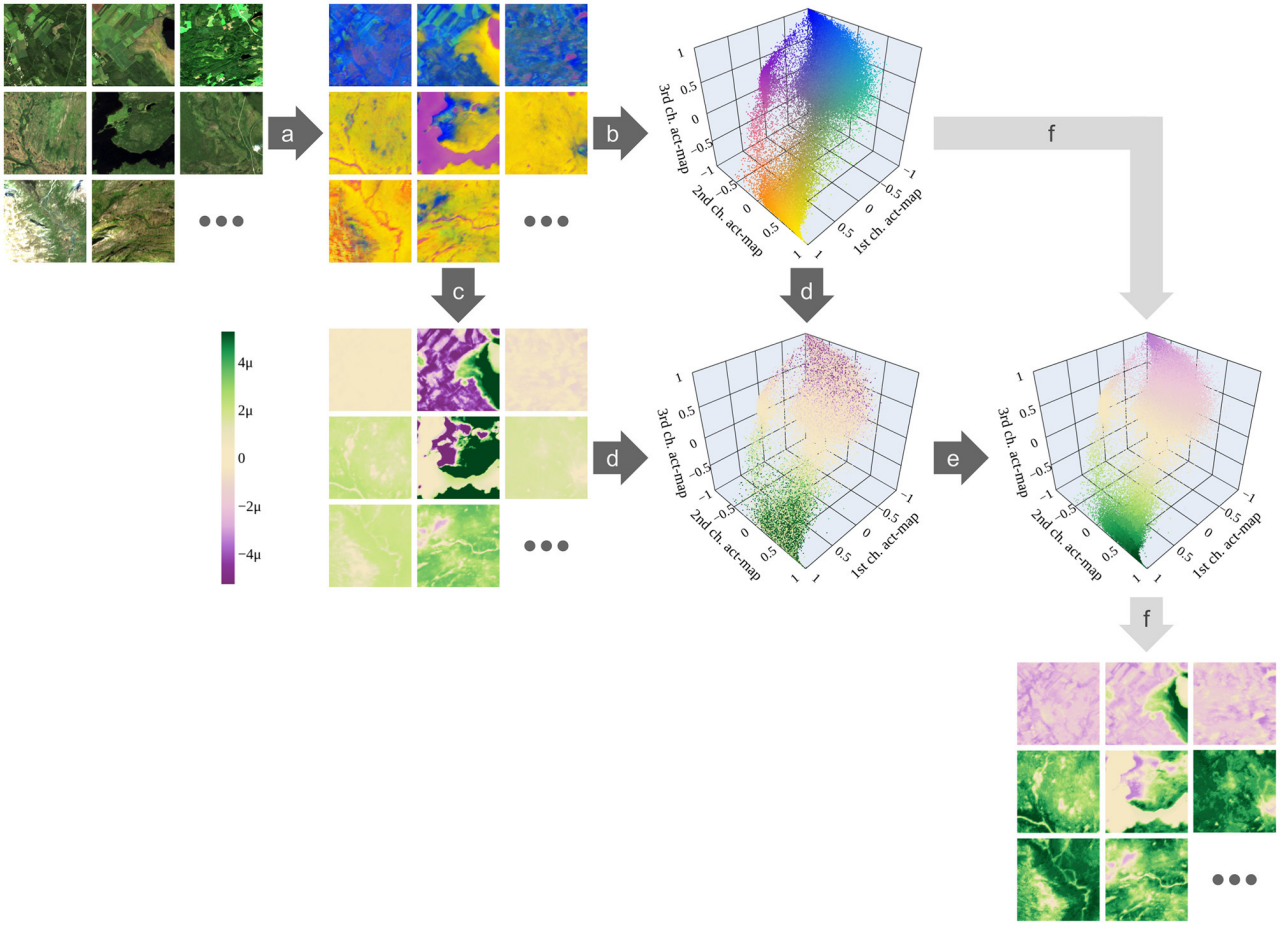
Harmonizing attributions. **(a)** Activation maps of all *N* = 19, 123 training images are predicted using the image-to-image network. Here, each activation map has *C*_act_ = 3 channels so that they can be visualized as red-green-blue images. **(b)** Each activation (pixel) in all activation maps is represented within the three-dimensional activation space, in which each axis represents the values of one of the three channels. **(c)** For each activation map, the attributions are determined—here using Grad-CAM. **(d)** All vectors in the activation space can be colored accordingly. **(e)** The mean values within the hypercubes are calculated. **(f)** Having used the training data, we have defined a linkage between activations and harmonized attributions. This linkage is used to directly determine attribution maps from activation maps—also for test data. The higher the attribution, the more the corresponding activation is connected with protected characteristics (green); the lower, the more it is connected to anthropogenic characteristics (purple); the closer to zero, the less connected to either of the classes (beige). In the color bar, μ stands for 1e-6. Only one out of 10,000 vectors is randomly chosen for visualizing the activation space. [Plotly Technologies Inc. (2015); Copernicus Sentinel data 2020].

We harmonize the attributions across the whole training set by calculating mean attributions for similar activations of the intermediate layer. To this end, we define an activation space as follows: Having *N* training samples, we obtain *N* activation maps at the intermediate layer. We treat each activation (pixel) in each activation map as a vector representing the *C*_act_ channels of the activation map. These activations build an *C*_act_-dimensional activation space, in which each axis represents the values of one of the *C*_act_ channels. The described steps are visualized in Figure 3, steps (a) and (b), for the specific case *C*_act_ = 3. The intuition for building the activation space is that similar activations are close together in this space.

Each activation (pixel) of all activation maps is represented as a vector in the activation space [Figure 3, steps (c) and (d)]. Hence, every vector has an attribution determined by the attribution method. We tile the activation space into ${\left(l_{\text{h}}\right)}^{C_{\text{act}}}$ hypercubes with a side length *l*_h_. Within each hypercube, we receive a distribution of attributions coming from similar activations. We define the mean value of these attributions to be a measure of the harmonized attribution for the volume of the hypercube [Figure 3, step (e)]. In this way, the activation space is discretized and an attribution is assigned to each discrete volume (hypercube).

Simply calculating the mean value over all attributions within a hypercube gives greater importance to data samples in which the corresponding activations are represented more often; and it gives less importance to those samples with fewer corresponding activations. Therefore, we calculate the mean attribution within each image and then take the unweighted mean of these mean attributions over all images.

#### 3.2.1 Activation space occlusion sensitivities (ASOS)

To obtain conventional occlusion sensitivities, rectangular-shaped patches are occluded in the activation maps. This might lead to simultaneous occlusions of different activations and, therefore, different high-level features. If these features contrast in their attribution, this leads to an inaccurate estimate of attributions. We, therefore, propose to not define the occlusions by patches in the activation maps, but by hypercubes in the activation space. For each hypercube, the activations within its volume are occluded in the activation map. This results in only similar activations being occluded at the same time.

### 3.3 Predicting attribution maps of test data

Attributions of test data are no longer determined using the original attribution method and the task-specific head. First, the activation map is predicted using the trained image-to-image network. Second, this activation map is evaluated using the linkage between activation values and harmonized attributions defined by the volumes of the hypercubes within the activation space. Since our image-to-image network is a pure CNN, test images can have any height *h* and width *w*.

Activations within low-density regions in the activation space rarely occur in the training data and are therefore not well represented by it. As a consequence, we set a density threshold to disregard these regions when predicting attribution maps and mask these areas (Figure 1F). This idea is related to the “area of applicability” estimation proposed by Meyer and Pebesma (2021) which, however, considers input features and distances to clusters instead of activations and densities, respectively. The exclusion of specific features increases the reliability of the other attributions.

### 3.4 Code availability

The code for the methodology and the experiments is available at: https://gitlab.jsc.fz-juelich.de/kiste/asos.

## 4 Study area and data

### 4.1 Protected and anthropogenic regions in Fennoscandia

Over the last 300 years, the landscapes of Fennoscandia (Norway, Sweden, Finland), especially forests, have seen anthropogenically-driven changes before regulations were introduced to protect them. This affects the southern regions more than the northern ones. Kouki et al. (2001) and Östlund et al. (1997) give detailed overviews of forest fragmentation in Fennoscandia and the transformation of the boreal forest landscape in Scandinavia, respectively. Nevertheless, there have been longstanding, strict conservation efforts in certain protected regions. All three countries have high environmental standards according to the Environmental Performance Index by Wendling et al. (2020). Furthermore, both the human influence index by Sanderson et al. (2002) and the wilderness quality index by Fisher et al. (2010) map wide areas within Fennoscandia as areas with relatively minimal (disruptive) anthropogenic influence.

To find protected territories, we use the World Database on Protected Areas (WDPA) by UNEP-WCMC and IUCN (2021) which contains polygons of protected areas categorized as proposed by Dudley (2008). We consider terrestrial areas of categories Ia (strict nature reserve), Ib (wilderness area), and II (national park) with a minimum area of 50 km^2^. To find anthropogenic areas, we use the Copernicus CORINE LC dataset by the European Environment Agency (2018) and locate areas with LC classes 1 (artificial surfaces) and 2 (agricultural areas). Then, three morphological functions are applied in the following order: (1) closing with a radius of 2 km to remove holes and gaps, (2) opening with a radius of 2 km to increase compactness and filter small structures, and (3) dilation with a radius of 1 km to create a buffer. Finally, all areas are filtered for a minimum area of 50 km^2^.

### 4.2 Multispectral Sentinel-2 imagery

Within the given regions, we export multispectral images of the Sentinel-2 satellites with a resolution of 10 m and a size of 256 × 256 pixels. Their instruments are specialized in vegetation which we assume to provide significant patterns of protection and anthropogenic influences, respectively. We decide to use Sentinel-2 over Landsat 8/9, due to the better spatial resolution and the additional red-edge bands. Further, Astola et al. (2019) conclude in a study that Sentinel-2 outperforms Landsat 8/9 in predicting forest parameters in Finland.

**Figure 4:**
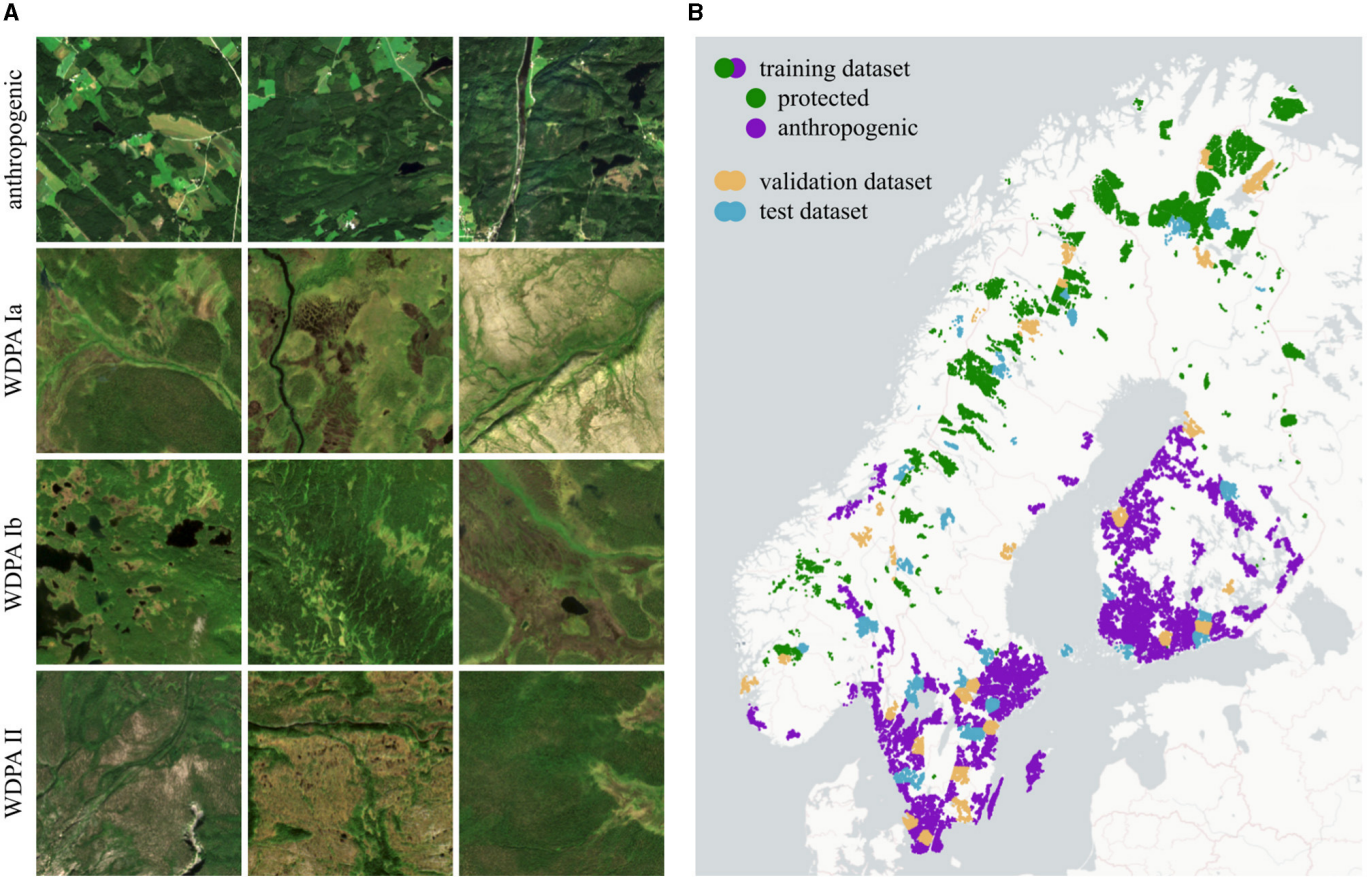
AnthroProtect dataset. **(A)** Shown are three Sentinel-2 images for each category: anthropogenic, WDPA Ia, Ib, and II. **(B)** Shown are the locations of the 7,003 protected and 16,916 anthropogenic samples which are split into three independent subsets for training (80%), validation (10%), and testing (10%). For better clarity, the coloring of both classes differs only for the training set. [Copernicus Sentinel data 2020; Plotly Technologies Inc. (2015); Copyright holders of the map are Carto and OpenStreetMap contributors].

For each protected and anthropogenic area, we proceed as follows: (1) The atmospheric corrected Sentinel-2 products (Level-2A) are used with a resolution of 10 m. Bands with a resolution of 20 m are upsampled using nearest-neighbor interpolation. (2) Images are filtered for the time period of summer 2020 (July 1st–August 30th). The temporal differences to WDPA from 2021 and to CORINE LC from 2018 have a negligible effect. (3) A mask for clouds, cirrus, and cloud shadows is created for each image using the Quality-60 m band (QA60) and scene classification map (SCL) provided by Sentinel-2. Only images with a mask fraction of <5% within the region of interest are taken. (4) The masked areas are dilated with a radius of 100 m to prevent artifacts at the transitions. (5) For each pixel and band, the 25th percentile is calculated across all remaining images in that region. Masked areas are not taken into account. This way, we receive a single image composite for that region. (6) The following ten bands are exported: B2, B3, B4, B5, B6, B7, B8, B8A, B11, B12. (7) We manually look at the red-green-blue channels (B4, B3, B2) and remove the image composite if it has strong artifacts. This concerns a total of two regions in the whole dataset. (8) The region of interest is tiled into images of size 256 × 256 pixels. Samples for each category are shown in Figure 4A.

Besides the mentioned protected and anthropogenic regions, some further Sentinel-2 images are exported for regions that are of interest for investigation. This includes several villages, forests, power plants, wind parks, airports, and more. For many of these regions, time series of the years 2017 to 2021 are included.

### 4.3 Land cover data

The following LC data is exported for each image and can be used for evaluation purposes: (1) the Copernicus CORINE LC dataset by the European Environment Agency (2018), (2) the MODIS LC Type 1 (Annual International Geosphere-Biosphere Programme Classification) by Friedl and Sulla-Menashe (2019), (3) the Copernicus Global Land Service by Buchhorn et al. (2020), (4) the ESA GlobCover by Arino et al. (2012), and (5) a composite (most common value) of the Sentinel-2 scene classification map (SCL). Data with lower resolution than 10 m has been upsampled using nearest-neighbor interpolation. The distribution of the CORINE LC classes over all protected and anthropogenic images in the dataset is listed in Table 1.

**Table 1:** Distribution of the CORINE LC classes in the AnthroProtect dataset separated by classes.

**CORINE LC class**	**Protected/%**	**Anthropogenic/%**
11 Urban fabric	0.0	2.4
21 Arable land	0.0	26.4
23 Pastures	0.0	1.0
24 Heterogeneous agricultural areas	0.0	8.2
31 Forest	34.6	50.8
32 Shrub and/or herbaceous vegetation associations	27.1	3.7
33 Open spaces with little or no vegetation	16.9	0.3
41 Inland wetlands	14.6	1.1
51 Inland waters	3.8	5.0
Other	3.0	1.1
	100.0	100.0

### 4.4 Data split

The data is divided into three subsets for training, validation, and testing with split fractions of 80/10/10%, respectively. The three subsets are independent of each other, spatially consistent, and categorically consistent. To ensure categorically consistency, the data split is performed separately for each category (WDPA categories Ia, Ib, and II, and anthropogenic). To ensure independence and spatial consistency, in the first step, spatial clusters are built as follows: Data samples are separated if their distance is larger than 10 km using the clustering algorithm DBSCAN developed by Ester et al. (1996). Large clusters are spatially clustered again, using the k-means algorithm by Lloyd (1982). Subsequently, all samples within one cluster are assigned to the same dataset. Hereby, samples within very small clusters are assigned to the training dataset. We use scikit-learn by Pedregosa et al. (2011) to perform both clustering algorithms. Compared with a random data split, our procedure reduces the incidence of nearby samples appearing in different datasets. The data split is listed in Table 2 and visualized in Figure 4B. It is ensured that all investigative regions do not overlap with samples of the training, validation, or test set.

**Table 2:** Number of samples (#) in the AnthroProtect dataset separated by categories and subsets.

**Class**	**WDPA category**	**# Train**	**# Val**	**# Test**	**# Total**
Protected	Ia	295	37	37	369
	Ib	3,601	465	446	4,512
	II	1,693	220	209	2,122
Anthropogenic	–	13,534	1,670	1,712	16,916
		19,123	2,392	2,404	23,919

### 4.5 Data availability

Our workflow for the data export is mainly based on Google Earth Engine by Gorelick et al. (2017). The full AnthroProtect dataset and the code for the data export are available at: https://phenoroam.phenorob.de/geonetwork/srv/eng/catalog.search#/metadata/6b1b0977-9bc0-4bf3-944e-bc825e466435.

## 5 Experiments and results

### 5.1 Main experiment

#### 5.1.1 NN and training

We build our model using PyTorch by Paszke et al. (2019). According to the multi-spectral Sentinel-2 data of our AnthroProtect dataset, the number of input channels is *C*_in_ = 10. We set the number of activation map channels to *C*_act_ = 3. Other values are possible which is addressed in Section 5.2. Our modified U-Net has about 1.8 million parameters. The task-specific head is significantly smaller with about 0.2 million parameters.

**Figure 5:**
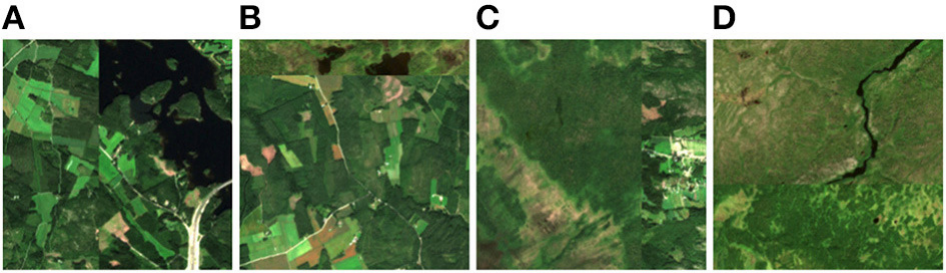
CutMix samples. **(A)** Two anthropogenic areas are CutMixed resulting in score 0. **(B)** An anthropogenic and a protected area are CutMixed resulting in score 0.16. **(C)** A protected and an anthropogenic area are CutMixed resulting in score 0.72. **(D)** Two protected areas are CutMixed resulting in score 1. [Copernicus Sentinel data 2020].

Our data provides a binary classification task with the classes anthropogenic and protected. However, we find that our methodology works best, if we define a regression task by implementing CutMix similar to Yun et al. (2019). This way, our NN acquires a continuous understanding of anthropogenic and protected characteristics making the NN sensitive to small changes which is useful for most attribution methods. When applying CutMix, we paste a stripe from a random sample to a random edge of the original image. Thus, a spatial relationship between the two categories (protected and anthropogenic) is maintained. The size of the stripe makes up a random amount between 0 and 50% of the original image. The label is adjusted proportionally resulting in a target score between 0 and 1. Samples of resulting images and their scores are shown in Figure 5. Yun et al. (2019) use CutMix as a data augmentation technique combined with the cross-entropy loss function. We, on the other hand, use CutMix to define a regression task from classification labels and, therefore, make use of the mean square error loss. Our last layer is activated with sigmoid, yielding predictions ŷ in the range of 0 (anthropogenic) to 1 (protected).

The pixel values of the Sentinel-2 Level-2A products range from 0 to 10,000. We normalize all values to be in a range from 0 to 1 and perform random image rotations of 0, 90, 180, 270° during training to increase variability. Our model is trained with a batch size of 32 and using all 10 available spectral bands. We perform the 1cycle learning rate policy by Smith and Topin (2019) with a maximum learning rate of 1e-2 and optimize the model's parameters with stochastic gradient descent. For regularization, we add a weight decay of 1e-4 to the loss function. In total, the model is trained for 5 epochs on a NVIDIA A100 40 GB PCIe for about 10 min.

**Figure 6:**
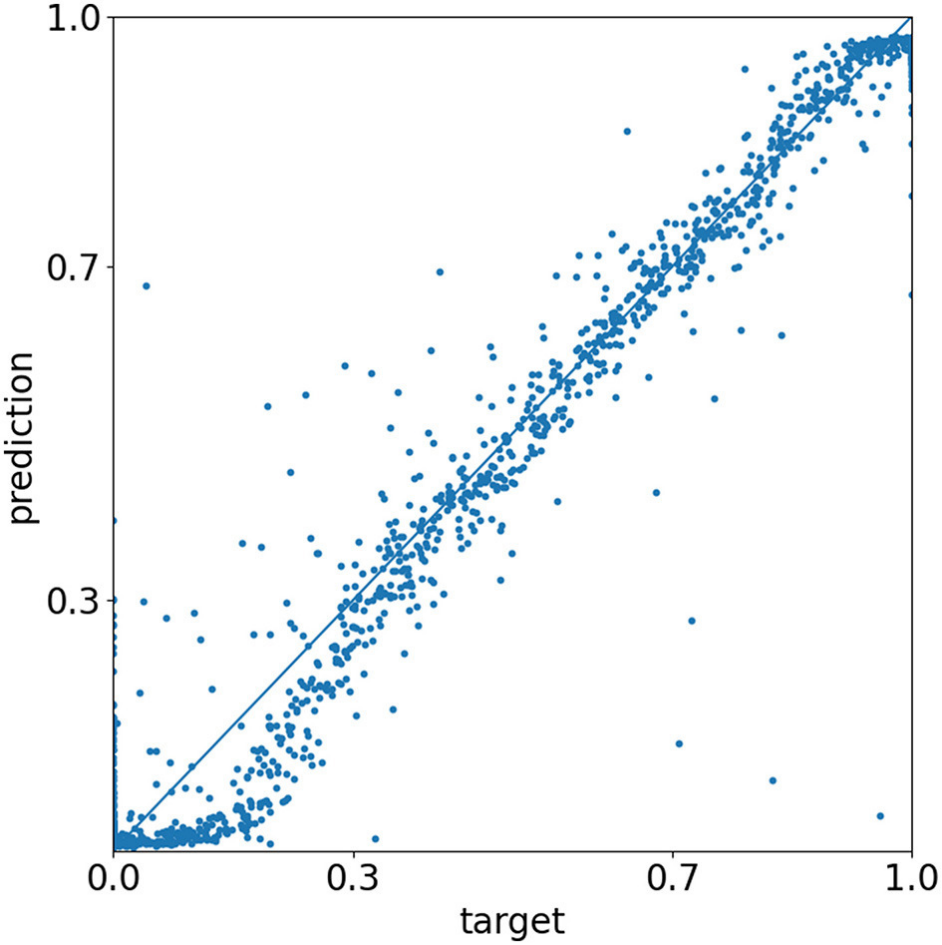
Evaluation of the test dataset. Each scatter point represents the prediction of a CutMixed image. The closer the point to the diagonal, the closer the prediction to the given target value. [Matplotlib by Hunter (2007)].

The normalized root mean square error calculates as $\sqrt{\text{MSE}}/\left(y_{\text{max}}-y_{\text{min}}\right)$, where MSE represents the mean square error, and *y*_min_ and *y*_max_ are 0 and 1, respectively. For the training, validation, and test datasets, it results in values of 5.7, 6.0, and 5.9%, respectively. Under the assumption of a normal distribution, this can be interpreted as the 1-sigma value, meaning that 68% of all predictions have an error lower than about 6%. The predictions of the test dataset are visualized in Figure 6. Predicting the non-CutMixed test samples with the same trained model and setting 0.5 as a decision threshold value, 1 out of the 1712 anthropogenic samples and 3 out of the 692 protected samples are falsely predicted.

#### 5.1.2 Attribution methods and harmonization

With our trained model, we predict *N* = 19, 123 activation maps for all CutMixed training samples. We determine their attributions by applying the two well-known attribution methods Grad-CAM by Selvaraju et al. (2020) and occlusion sensitivity mapping by Zeiler and Fergus (2014) to demonstrate our methodology. Both methods are computed using the Captum library by Kokhlikyan et al. (2020). We further apply our proposed ASOS method, which is based on occlusions defined by the hypercubes in the activation space (Section 3.2.1).

Usually, only positive attributions are considered when applying Grad-CAM by utilizing ReLU. However, since we face a single-target regression task, we do not restrict the attributions to positive values only. To determine occlusion attributions, we use a patch side length of *l*_patch_ = 8 and a stride of *l*_stride_ = 4. As discussed in Section 3.2, we cover the tanh-activated values with zeros to switch them off.

We use the activation space to harmonize the attributions as described in Section 3.2. We set the side length of the hypercube to *l*_h_ = 0.1 which results in 8,000 hypercubes across the activation space. We evaluate this number as a good compromise between continuity and computation time. The use of other numbers of hypercubes is discussed in Section 5.2.

#### 5.1.3 Comparing attribution methods

**Figure 7:**
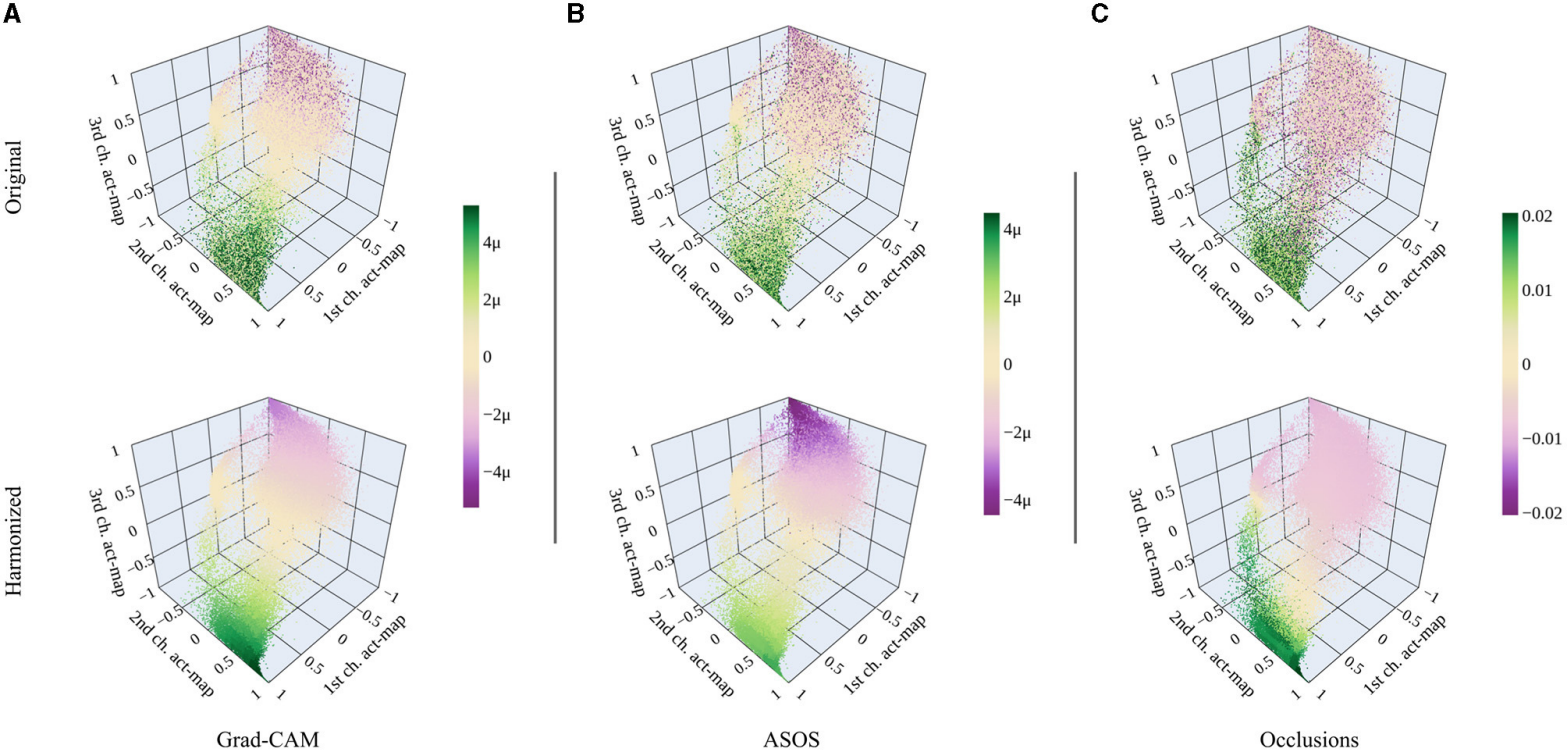
Original and harmonized attributions for different attribution methods. **(A)** Grad-CAM. **(B)** ASOS. **(C)** Occlusion sensitivity mapping. The range of the color bar is chosen according to the 99.9 percentile of all absolute harmonized attribution values for every method. [Plotly Technologies Inc. (2015)].

Figure 7 shows the original and harmonized attributions within the activation space for all tested attribution methods. For Grad-CAM and ASOS, positive and negative attributions are clearly separated across the activation space with neutral attributions in between. Such a neutral area does not exist for occlusion sensitivity mapping. Instead, original attributions are rather mixed in the corresponding area. Other than the first two attribution methods, occlusion sensitivity mapping works independently of the activation values. Square-shaped patches are occluded in the map and thus, various activations (features) might be occluded at the same time leading to more noise within the activation space.

After harmonizing the attributions, their distribution across the activation space is similar for all methods. There are some differences in the intensities, e.g., Grad-CAM's positive attributions are more intense while for ASOS it is the negative attributions. Further, the neutral area is shifted for the occlusion sensitivity mapping.

The similarity of the harmonized activation spaces consequently applies to the attribution maps. Therefore, we present only the results for Grad-CAM in the following.

#### 5.1.4 Analyzing test data

**Figure 8:**
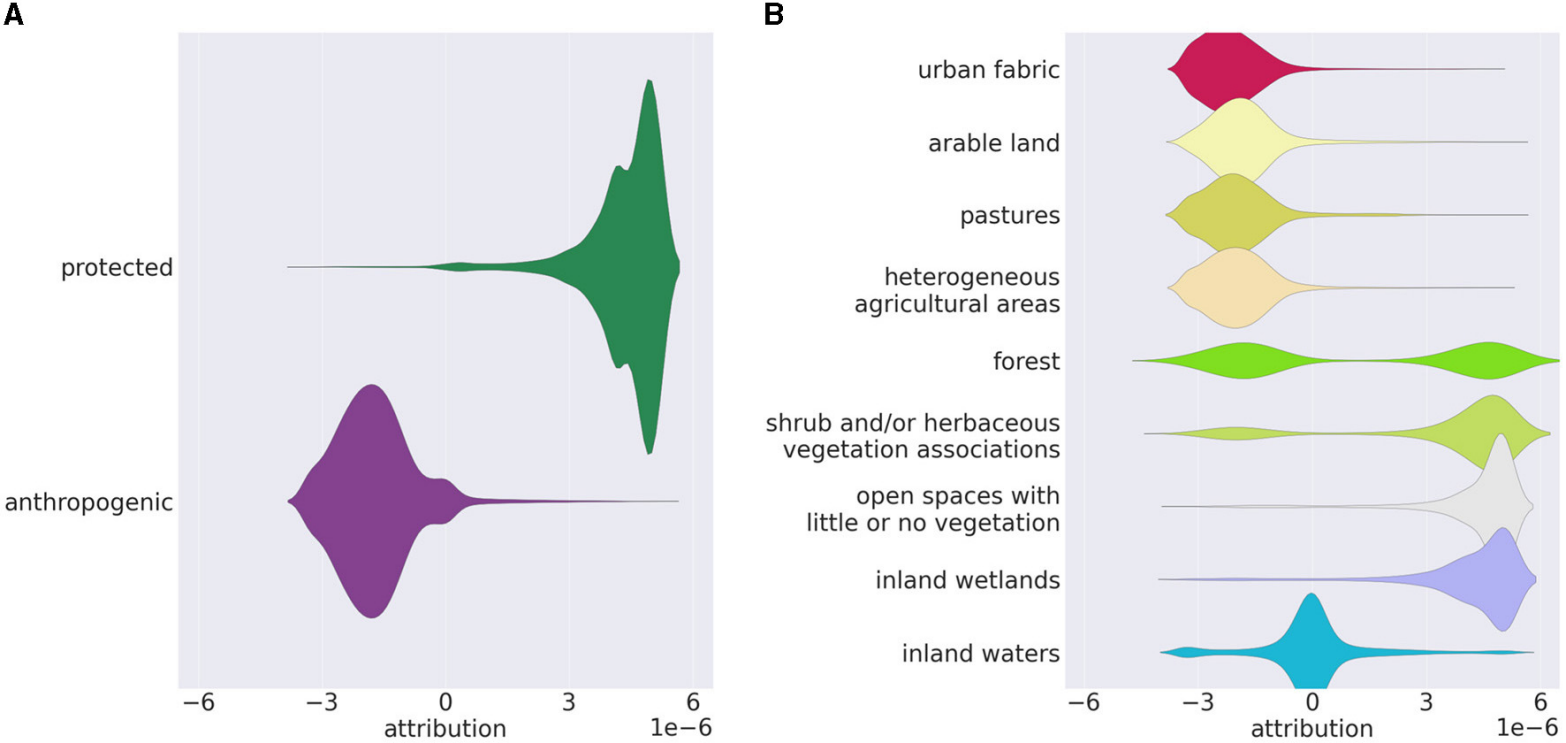
Distributions of the Grad-CAM attributions in the test dataset without CutMix, **(A)** subdivided into protected and anthropogenic regions; **(B)** subdivided into CORINE LC classes. The visualization compensates for an uneven amount of the subcategories within the dataset. [Seaborn by Waskom (2021); Matplotlib by Hunter (2007)].

Figure 8 illustrates some distributions of the harmonized attributions of the test dataset without CutMix. Scenes showing protected areas have almost entirely positive attributions, while scenes showing anthropogenic regions have mainly negative attributions (Figure 8A). Figure 8B shows the distributions of attributions subdivided into CORINE LC classes present in the test dataset. The distributions basically reflect the amounts of LC in the dataset classes (see Table 1): LC classes that only occur in anthropogenic areas (urban fabric, arable land, etc.) have almost entirely negative attributions; LC classes that mostly occur in protected areas (open spaces, inland wetlands) have almost only positive attributions. LC classes occurring in both areas (forest, shrubs, and herbaceous vegetation, etc.) have both negative and positive attributions. Patterns that clearly assign inland waters to one of the two classes hardly seem to exist, resulting in a distribution around zero.

#### 5.1.5 Analyzing scenes

**Figure 9:**
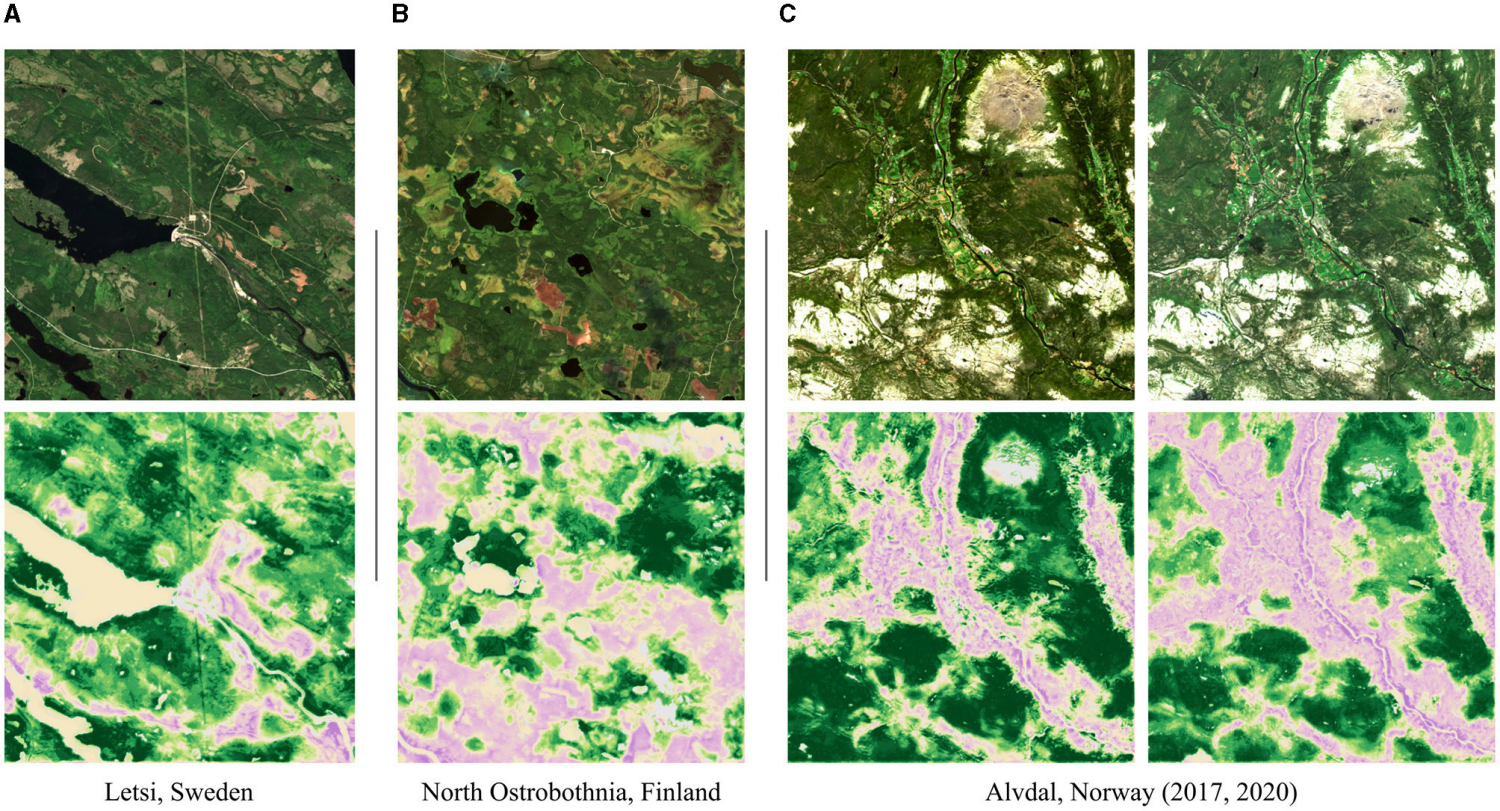
Scene samples showing a Sentinel-2 image and its harmonized Grad-CAM attribution map. **(A)** Hydroelectric power plant Letsi in Sweden, located in an area barely inhabited by humans. The shown scene covers about 100 km^2^. **(B)** Forestry and unclaimed wetlands encounter in North Ostrobothnia, a region of Finland. The shown scene covers about 100 km^2^. **(C)** Municipality Alvdal in Norway, whose villages are located within the Østerdalen valley. The scene covers about 420 km^2^. Between 2017 and 2020, there was a large anthropogenic expansion due to deforestation. The color scale of the attribution maps is the same as in Figures 3, 7. Attributions not predicted due to a low density in the activation space are colored in gray. [Copernicus Sentinel data 2017 and 2020].

The attribution maps of some representative scenes are shown in Figure 9. We mask pixels that have activations in a low-density region within the activation space of the training data (visualized in gray), specifically, a density <0.5 of the average density. The three scenes in Figure 9 show: (A) the hydroelectric power plant Letsi in Sweden which is located in an area barely inhabited by humans; (B) North Ostrobothnia, a region in Finland, in which forestry and unused wetlands encounter according to LUCAS data by Eurostat (2018); (C) the villages of the municipality Alvdal in Norway which are located within the Østerdalen valley. For the last example, there has been significant tree cover loss within the years 2017–2020 according to the global forest loss mapping by Hansen et al. (2013, https://gfw.global/3qJLGPX). Deforestation areas can be seen in the Sentinel-2 image of 2020 but appear very small. Based on the analysis of more examples, we make the following observations: (1) Anthropogenic influences such as villages and agricultural areas are well-mapped with our method (e.g., municipality Alvdal in 2017). So are large interruptions, e.g., caused by power plants (see power plant Letsi). (2) Deforestation areas are also well-detected. If there are single but large deforestation areas, the area is usually mapped accordingly (e.g., power plant Letsi, above the upper lake). If there are multiple small deforestation areas, attributions are spatially expanded over the whole fragmented area (municipality Alvdal in 2020). (3) Spatial expansions also occur along some roads (power plant Letsi, lower part). However, other roads might not be mapped at all (North Ostrobothnia). The same applies to most power lines (power plant Letsi, going centered from top to bottom). (4) Our model distinguishes between different patterns occurring in forests. For example, the forests in Letsi have protected patterns, whereas the forests in North Ostrobothnia have anthropogenic patterns. In fact, a closer look at the Sentinel-2 image of North Ostrobothnia (bottom left) reveals that trees have been planted in rows. (5) Wetlands have almost always protected attributions (North Ostrobothnia, top right). This behavior is so strong that even the brownish fields in the lower part of the image result in protected attributions. These fields, however, are peat production areas.

### 5.2 Complementary experiments

Unless specified, we use the same training procedure as for the main experiment in Section 5.1 including hyperparameter and model architecture choices.

**Figure 10:**
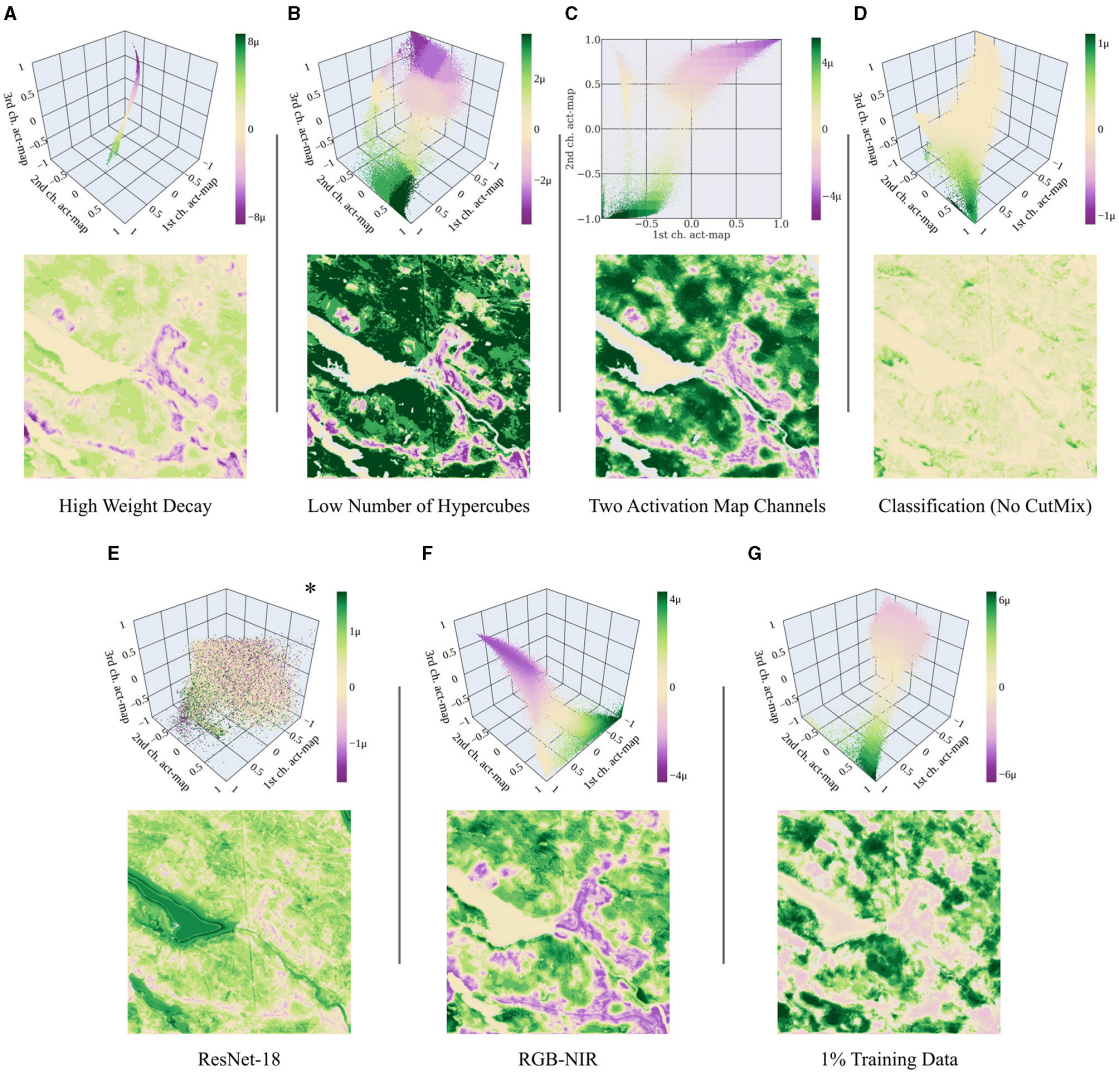
Harmonized activation spaces and attribution maps for: **(A)** High weight decay, specifically 1e-2 instead of 1e-4. **(B)** Low number of hypercubes, specifically 64 instead of 8000. **(C)** *C*_act_ = 2 activation map channels. **(D)** Classification task without CutMix instead of regression. **(E)** ResNet-18 as task-specific head instead of a shallow CNN. *Unlike the others, this activation space shows the non-harmonized attributions. **(F)** Using red, green, blue, and near-infrared channels only. **(G)** Using 1% of the training data corresponding to 191 samples. The range of the color bar is individually chosen according to the 99.9 percentile of all absolute harmonized attribute values. The results of the main experiment are shown in Figures 7, 9. [Plotly Technologies Inc. (2015); Matplotlib by Hunter (2007)].

**Weight decay:** A relevant parameter that affects the appearance of the activation space is the weight decay. The higher the weight decay, the denser the activations in the activation space. A large weight decay leads to a collapse of activations into a narrow, tube-like area (see Figure 10A). However, this does not significantly change the harmonized attribution maps. Although the intensities change, the basic appearance remains similar.

**Number of hypercubes:** The attributions are harmonized by subdividing the activation space into hypercubes. The number of hypercubes does not directly influence the spatial resolution of the attribution maps as they group the activations according to their values rather than their spatial location. Minor differences can be observed in the transitions between the harmonized attributions. With fewer hypercubes, the transitions become less smooth and more abrupt (see Figure 10B).

**Number of activation map channels:** In the main experiment, we present results obtained with *C*_act_ = 3 activation map channels, resulting in a three-dimensional activation space. If *C*_act_ = 2, the activation space can be visualized on a plane; if *C*_act_ = 1, it can be visualized as a histogram. The number of possible hypercube positions exponentially grows with the number of dimensions, making the attribution analysis more computationally expensive for higher dimensions. Although the mean square error of our NN is similarly high when choosing *C*_act_ = 1, the resulting attribution maps look abstract, e.g., containing stripe-like structures. If we choose *C*_act_ = 2, the resulting 2-dimensional activation space looks similar to a projection of the three-dimensional one onto a plane (see Figure 10C). Thus, the attribution maps look very similar and it makes little difference whether we choose *C*_act_ = 2 or *C*_act_ = 3.

**Regression vs. classification task:** In Section 5.1, we train our model on a regression task by implementing CutMix. Training our model using classification labels and the binary cross-entropy loss, the negative and positive attributions have significantly different intensities (see Figure 10D). The reason for the different behavior is that for classification, a small part within an image might have a rare effect on the overall decision which has a corresponding effect on the attributions. Except for the intensities, we nevertheless obtain similar results in the attribution maps.

**ResNet-18 as task-specific head:** We replace the small task-specific head of our NN with a ResNet-18. This way, the intermediate layer, to which the attribution methods are applied, is no longer at the rear part of the NN. Since this NN is significantly deeper, we train it twice as long, namely 10 epochs. Positive and negative attributions mix significantly more within the activation space (see Figure 10E).

**RGB-NIR bands:** We evaluate how a change in the input channels affects the appearance of the activation space. Training our model using only bands B2 (blue), B3 (green), B4 (red), and B8 (near infrared) yields similar results compared to our model trained with all bands (Figure 10F). Also, the appearance of the activation space is similar to the original space, albeit rotated and more peaky. To run this experiment, we modify the number of input channels of the model to *C*_in_ = 4 instead of 10.

**Training data size:** We further evaluate how the amount of training data affects our results and use a random subset of 1% of the training data corresponding to 191 samples. We train the model for 500 epochs instead of 5 to maintain the total number of iterations. The results show that the activation space and the attribution maps appear similar to the ones obtained with the full training dataset (Figure 10G).

## 6 Discussion

In the field of remote sensing, it is crucial that results are comparable within large scenes and across different scenes. However, this is generally not the case for attribution methods because multiple patterns within a single image interact and influence each other (see Figure 1D). Fortunately, inputs and activations have a consistent relationship in pure CNNs, if we disregard the outer edges of the image. By harmonizing the attributions across the training data, we further create a linkage between activations and attributions. Harmonized attribution maps are thus consistent across inputs and remote sensing scenes can be evaluated adequately.

The activation space allows for analyzing and evaluating the distribution of attributions in relation to the activations at a glance across a dataset. For our task, we observe a reasonable assignment for all tested attribution methods: Positive and negative attributions are separated within the activation space and there is a transition between both areas (cf. Figure 7). This behavior strengthens confidence in the original attribution methods. Excluding low-density regions from the prediction of attribution maps further increases reliability.

Using several attribution methods often results in significantly different attribution maps (e.g., Kakogeorgiou and Karantzalos, 2021). However, by harmonizing the attributions we obtain similar outcomes for all tested attribution methods (cf. Figure 7). Certainly, the intensities of attributions and the region of neutral attributions within the activation space slightly differ. Basic behavior, however, remains the same. To adjust those differences, in future research, our method can be used to harmonize multiple attribution methods by averaging them within the activation space. This can help leverage the strengths of different attribution methods and balance their weaknesses. Furthermore, the robustness of the interpretation can be analyzed this way.

Our approach fulfills two aspects of weakly-supervised learning, namely, inexact and inaccurate supervision (Zhou, 2018); inexact because we do not provide segmentation data, and inaccurate because there might be anthropogenic influences in protected areas and vice versa within the training data. This makes our methodology promising for weakly-supervised segmentation learning (Pathak et al., 2015; Kwak et al., 2017) including multi-class problems. Here, attributions are vectors and can be harmonized target by target so that one obtains one attribution map for each target. Theoretically, this works with any kind of gridded data, including photographic images. However, our methodology is primarily designed to recognize patterns in test scenes associated with certain properties rather than to predict exact segmentation boundaries. In photographic images, these patterns are often edges; hence, applying our method for weakly-supervised segmentation is only meaningful to a limited extent. For LC classes in remote sensing images, the crucial patterns are often structures; thus, the application of weakly-supervised segmentation learning is more appropriate. Nevertheless, the spatial expansion of the images' context is not preserved in activation maps of deeper layers. That is naturally given due to convolutional and pooling calculations and has an equivalent effect on the attributions. Harmonizing the attributions cannot enhance this issue. For this reason, very fine objects like roads are not suitable for exact segmentation purposes. Using an Attention U-Net as proposed by Schlemper et al. (2019) instead of a pure CNN U-Net might help in improving this issue since attention layers help to focus the activations on certain spatial regions in the image. In future research, it can even be investigated whether attentions can be harmonized meaningfully instead of activations.

There are many types of human influence and not all of them are apparent from a satellite's perspective. For example, anthropogenic influence on wildlife cannot be detected unless the wildlife has a measurable effect on the vegetation. Plants or soil obscured by larger plants are not apparent in satellite images. ML models generally show a low generalization ability if the test data has a significantly different distribution compared to the training data. Thus, outside of Fennoscandia, our trained model is likely not applicable—particularly if the region has distinctly different landscapes or ecosystems such as savannas or tropical forests. Here, one could set up a training dataset similar to AnthroProtect for the biogeographic region(s) of interest. However, the low density of strictly protected or wilderness areas in some biogeographic regions might make it challenging to create a suitable dataset.

## 7 Conclusion

In this article, we present an approach for mapping land cover patterns of protected and anthropogenic areas using interpretable ML techniques. Specifically, we propose a novel mechanism to accordingly map these patterns considering a learned representation that captures high-level features in full resolution. We do not explain the model's behavior for single input images, as it is commonly done, but we average the overall behavior across the training dataset to recognize the existing patterns. This is realized by establishing a direct relationship between activations and attributions, ensuring consistency across different scenes, and enhancing the trustworthiness and reliability of the results. Our methodology allows for seamless integration with a wide range of attribution methods, including Grad-CAM and occlusion sensitivity mapping. At the same time, it enables a novel attribution technique: activation space occlusion sensitivities (ASOS).

Territorial protection can offer important ecological and social benefits; and there are urgent and pragmatic reasons (conservation, sustainable development, etc.) to identify where the positive characteristics and ecological functions associated with naturalness are present and able to flourish. With this objective, satellite imagery allows for continuous monitoring of broad, undisturbed regions without the need of visiting them. Being able to analyze the extent and characteristics of non-anthropogenic areas, even if approximate, is a valuable tool that could be utilized for tracking conservation areas, reforestation efforts, and existing ecological naturalness.

## Data availability statement

The dataset presented in this study can be found in an online repository. The name of the repository and accession number can be found below: The AnthroProtect dataset and the code for the data export are available at https://phenoroam.phenorob.de/geonetwork/srv/eng/catalog.search#/metadata/6b1b0977-9bc0-4bf3-944e-bc825e466435. The code for the methodology and the presented experiments are available at: https://gitlab.jsc.fz-juelich.de/kiste/asos.

## Author contributions

TS: Conceptualization, Data curation, Formal analysis, Investigation, Methodology, Software, Validation, Visualization, Writing—original draft, Writing—review & editing. JL: Data curation, Methodology, Writing—review & editing. IW: Methodology, Writing— review & editing. RR: Conceptualization, Methodology, Supervision, Writing—review & editing.
